# Population Genetic Structure and Post-Establishment Dispersal Patterns of the Red Swamp Crayfish *Procambarus Clarkii* in China

**DOI:** 10.1371/journal.pone.0040652

**Published:** 2012-07-10

**Authors:** Yanhe Li, Xianwu Guo, Xiaojuan Cao, Wei Deng, Wei Luo, Weimin Wang

**Affiliations:** 1 College of Fisheries, Key Laboratory of Agricultural Animal Genetics, Breeding and Reproduction of Ministry of Education, Huazhong Agricultural University, Wuhan, People’s Republic of China; 2 Institute of Fisheries, Anhui Academy of Agricultural Sciences, Hefei, People’s Republic of China; 3 Laboratorio de Biomedicina Molecular, Centro de Biotecnología Genómica, Instituto Politécnico Nacional, Boulevard del Maestro esquina Elías Piña, Colonia Narciso Mendoza, Tamaulipas, Mexico; Auburn University, United States of America

## Abstract

The red swamp crayfish (*Procambarus clarkii*) was introduced to China in the early 20^th^ century. It has been spread to almost all forms of fresh water bodies including lakes, rivers and even paddyfields in most provinces of China. To clarify issues such as the initial entry point(s), dispersal pattern, genetic diversity and genetic structure of *Procambarus clarkii* in China, the genetic structure and diversity of *P. clarkii* populations at 37 sampling sites (35 from China, one from the USA and one from Japan) were analyzed using both mitochondrial gene sequences (COI and 16S rRNA) and 12 nuclear microsatellites. Multiple tests including phylogenetic analyses, Bayesian assignment and analysis of isolation by distance showed that (i) the population from Japan and those collected from China, particularly from NanJing (BGt and XG) and its some neighboring sites (CJr, NT and NB), have similar genetic composition, (ii) relatively high genetic diversity was detected in Chinese populations, (iii) the *P. clarkii* populations in China did not experience significant population expansions. Taken together, Nanjing, Jiangsu province is the presumed initial entry point, and human-mediated dispersal and adaptive variation are likely responsible for the observed genetic pattern of *P. clarkii* in China.

## Introduction

Successful biological invasions require that non-indigenous species pass through a series of filtering stages including transport, release, population establishment, and in many cases, dispersal. A successful invader is characterized by a number of biological and ecological features for its dispersal and establishment in a new habitat [1]. The red swamp crayfish (*Procambarus clarkii*), native to south central USA and northeastern Mexico, is one of the most notorious invasive species in the world [2], [3]. Compared to some invasive animals such as insects, the natural dispersal capacity of *P. clarkii* is relatively weak [4]. However, anthropogenic activities are considered to play a crucial role in translocation of the red swamp crayfish [5]. Furthermore, high fecundity, short development time and flexible feeding habits provide this species a high adaptability to various ecosystems. A well documented example of rapid expansion is the dispersal of this crayfish in the southern Portugal and Mediterranean wetlands [4], [6]. Since this crayfish was introduced into two aquaculture installations located in Sevilla and Badajoz in Spain [7] in 1973, it has become a widespread species throughout southern Portugal, the Mediterranean wetlands and some other places in Europe only for three decades [4], [6], [8].

*P. clarkii* was introduced to Nanjing, China from Japan in 1929 [9]. Nowadays this species has been found in almost all forms of fresh water bodies including lakes, rivers and even paddyfields in most provinces of China [10]. Because of its high commercial value [10], *P. clarkii* aquaculture has been developed rapidly, and this species has become one of the most important aquatic products in China [11]. Presently, much attention is being paid to technologies of culture, reproduction and breeding of this species, but less attention is being given to its invasions and related ecological and ecological effects, let alone the invasion genetics of *P. clarkii*. In general, the red swamp crayfish threatens local biodiversity in freshwater ecosystems, and their burrows result in dam damages and a huge loss of irrigation water, causing significant economic loss [12], [13]. In addition, *P. clarkii* is considered as an important pest of wet-seeded rice (*Oryza sativa*) fields [14].

Information on the population structure changes would help to understand the biological invasions of this alien species, and in some cases, would be useful for the establishment of possible methodologies for prevention and control of its invasions [15]. A study of the species population structure and genetic diversity would provide valuable data to clarify issues such as the initial entry point(s) and dispersal pattern of *P. clarkii* in China. A few articles have touched upon these topics to date [5], [9], [10], however, no systematic genetic data from microsatellite loci and mitochondrial DNA sequences have yet been generated to address such issues.

In this study, we aim to clarify the issues including: (i) whether the *P. clarkii* populations in China have relatively high genetic diversity, which would likely facilitate its invasion success to some extent, (ii) whether Nanjing was the initial point of entry of *P. clarkii* in China, (iii) whether the introduction of *P. clarkii* in China was derived from a single or multiple event(s). Here we used both mitochondrial gene sequences (COI and 16S rRNA) and 12 nuclear microsatellites to clarify dispersal pattern, genetic diversity and genetic structure of *Procambarus clarkii* in China.

## Results

### Genetic Diversity

The number of alleles ranged from three to 27 across all 12 microsatellite loci in 37 populations (*N* = 1776). The mean observed heterozygosity (*Ho*), the mean expected heterozygosity (*He*), and the polymorphism information content (*PIC*) were 0.6723, 0.7913 and 0.7551, respectively. The Lo (Louisiana) and Sa (Saitama) population exhibited the highest genetic diversity among the 37 populations, while the ZX population showed the lowest (Table S1). All the *P. clarkii* populations in China showed relatively high genetic diversity: number of alleles (*Na*) = 6.4–11.8, and expected heterozygosity (*He*) = 0.7002–0.8214. Deviations from HWE were observed at multiple loci in multiple populations. Most of the deviated cases showed significant heterozygote deficiency.

Of the 313 individuals examined using the COI gene, the nucleotide diversity was 0.0022. Only six haplotypes (GenBank accession numbers: JX120103-JX120108) were found. There were some base substitutions but no insertion or deletion was found. Twelve parsimony informative sites and three singleton variable sites were detected (Figure S1A). The haplotype diversity of the partial COI sequence (*Hd*) was 0.403. The variance and the standard deviation of haplotype diversity was 0.00072 and 0.027, respectively. Under the Maximum Composite Likelihood model, the overall mean pairwise genetic distance of the six haplotypes was 0.011. Most individuals (231/313) had Hap_C2, which was mainly distributed among the *P. clarkii* populations collected in China and Japan. The haplotypes Hap_C3, Hap_C4, Hap_C5 and Hap_C6 were only detected in the population collected in the USA (Figure S2A).

Based on the data of partial 16S rRNA sequences (284 individuals), the nucleotide diversity was 0.00083. Only three haplotypes (GenBank accession numbers: JX120109- JX120111) were found. Two parsimony informative sites and one base deletion were detected (Figure S1B). The haplotype diversity (*Hd*) was 0.390, while the variance of haplotype diversity was 0.00077 and its standard deviation was 0.028. Under the Maximum Composite Likelihood model, the overall mean pairwise genetic distance of the three haplotypes was 0.003. Seventy-five percent of the examined individuals (212/284) had Hap_S2, which was mainly distributed among the populations collected in China and Japan. The Hap_S3 was only detected in the population collected from the USA, whereas the Hap_S1 was shared among in the populations collected in China, Japan and the USA (Figure S2B).

### Analyses of Population Structure and Assignment Tests

Based on the microsatellite genotype data, the Nei's genetic distance (0.145) between the populations XYw and XYc was the lowest, while the Nei's genetic distance (0.999) between the populations ZX and Sa was the highest. The gene flow parameter among populations ranged from 1.284 between the populations DY and PYL to 10.595 between the populations XYw and XYc. The gene flow between the populations XYw and XYc occurred very frequently and its parameter value was far greater than those among other populations (range from 1.284 to 6.968). The coefficient of genetic differentiation (*Fst*) in 37 populations of *P. clarkii* ranged from 0.023 between the populations XYw and XYc to 0.157 between the populations DY and YJ, NX (details not presented).

**Figure 1:**
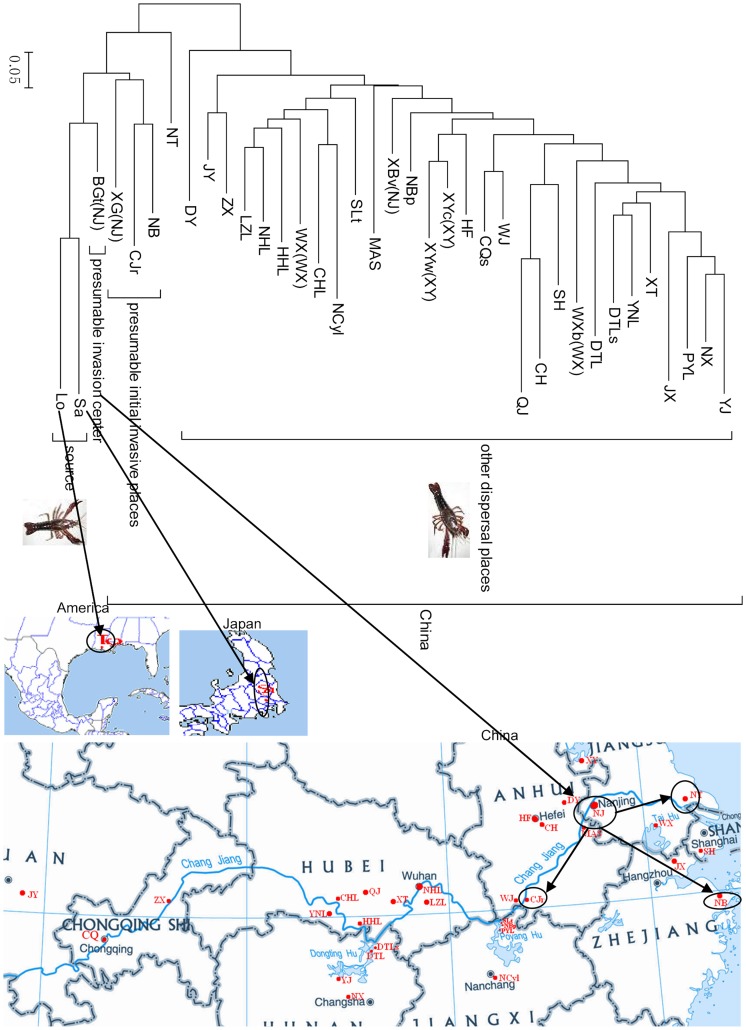
Sampling locations of *P. clarkii* and their populations cluster analysis. Red solid dots on the maps indicated the sampling locations. The blue line in China maps denoted the Changjiang River. XY includes XYw and XYc population. NJ includes XG, BGt and XBv population. WX includes WX and WXb populations. What the sampling location codes in the figure indicated sees Table S1.

The neighbour-joining tree consisted of two major clades. One major clade included the populations of *P. clarkii* collected in Japan and the USA (Lo and Sa), and some Chinese populations. The other big clade included all the remaining *P. clarkii* populations collected in China (Figure 1).

**Figure 2:**
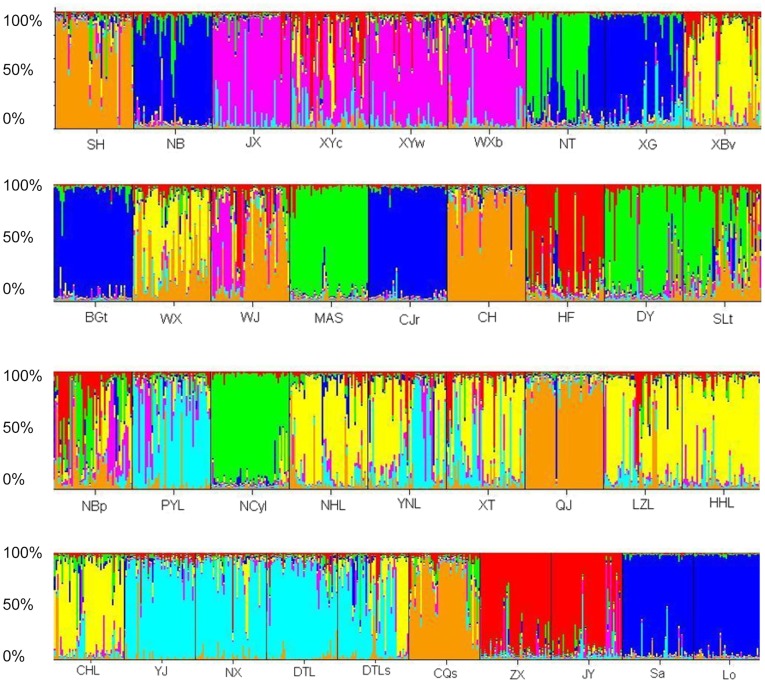
Structure version 2.3.1 analysis of *P. clarkii* populations using microsatellite genotype data from twelve microsatellite loci (the inferred clusters, K = 7). The sampling location codes (see Table S1) are indicated along the X-axis. Each vertical line represents one individual, and Y-coordinate denotes each individual’s percentage assignment to each of these seven genetic clusters.

Genetic clustering analysis using Structure program [16], [17] indicated that the number of genetic clusters was seven (*K* = 7; Figure 2). Most individuals from four Chinese populations (XG, BGt, NB and CJr) and two foreign populations (Sa and Lo) were assigned to the same genetic cluster, suggesting that they might share the same origin (represented by dark blue in Fig. 2), while some individuals of sampling sites in China tended to be more admixed (such as XYc, WX, WJ, HF, SLT, Fig. 2).

### Population Differentiation and Spatial Genetic Structure

The Analysis of molecular variance (AMOVA) of microsatellite genotype data revealed that 91.26% of genetic variation could be explained by the variation within populations, whereas the remaining (8.74%) came from variation among populations (Table 1). However, the AMOVA of mitochondrial COI sequences analysis revealed that 45.67% of genetic variation could be explained by the variation within populations, whereas the remaining (54.33%) came from variation among populations. Meanwhile, the AMOVA of mitochondrial 16S rRNA sequences analysis revealed that 43.32% of genetic variation could be explained by the variation within populations, whereas the remaining (56.68%) came from variation among populations.

**Table 1:** Analysis of molecular variance within and among *P. clarkii* populations by microsatellite, mtDNA COI and 16S rRNA analysis.

Source of variance	d.f.	Sum of squares	Variance components	Percentage of variation (%)
microsatellite analysis				
Among populations	36	1702.299	0.44426	8.74
Within populations	3515	16300.219	4.63733	91.26
Total	3551	18002.517	5.08159	
mtDNA COI analysis				
Among populations	36	130.401	0.38973Va	54.33
Within populations	276	90.436	0.32767Vb	45.67
Total	312	220.837	0.71740	
mtDNA 16S rRNA analysis				
Among populations	36	68.521	0.22555Va	56.68
Within populations	247	42.577	0.17238Vb	43.32
Total	283	111.099	0.39792	

Regressions of genetic distances against geographical distances were not significant (Mantel test: microsatellite genotype, r = 0.052, *P* = 0.710; mitochondrial COI sequences, r = 0.036, *P* = 0.688; mitochondrial 16S rRNA sequences, r = 0.043, *P* = 0.631). Thus, the crayfish in China did not show isolation by distance.

### Analyses of Population Expansion

The detection of population expansion was performed using *Fu* and *Li’s* Tests. The positive *Fs* values were 1.861 (P = 0.256>0.05), derived from mitochondrial COI sequences analysis and 0.860 (P = 0.567>0.05) from mitochondrial 16S rRNA sequences analysis, indicating that the *P. clarkii* did not significantly experience the population expansion.

## Discussion

### Invasion Population Structure and Dispersal Pattern

Several reports have suggested that natural dispersal between rivers or/and lakes had the major impacts on the dispersal of *P. clarkii* [5], [10]. Based on our genetic analyses, the rates of gene flow among population BGt and other populations along the Changjiang (Yangtze) River, such as WX, MAS, and CJr were higher than that among the populations (BGt, XG and XBv) in Nanjing and two Xuyi populations, which also indicated the importance of natural dispersal between rivers or/and lakes for *P. clarkii* dispersal. However, most of the *P. clarkii* populations in China are separated by geographic barriers (such as land, mountains and the Yangtze River), which could be major physical barriers to natural dispersal. However, we detected a high level of gene flow among populations separated by geographic barriers. For example, the gene flow between populations NBp and HF was 5.489 (Figure 1). Moreover, the genetic distances among the populations were not significantly correlated with geographic distances for *P. clarkii* populations. Collectively, human mediated-dispersal, especially aquaculture transfers, could be responsible for the observed genetic patterns. Dispersal of aquatic exotic species in nature ecological systems has often been influenced by human mediated vectors [18], [19], [20], especially in fresh water ecological systems [15], [21]. As *P. clarkii* is an economic aquaculture species in China, frequent aquaculture-related transfers could mediate dispersal among different locations [9]. Thus the population genetic structure presented here likely resulted from a combination of natural expansion and human-mediated jump dispersal. Human activities such as commercial shipping or unintentional carry have probably facilitated communication among populations of *P. clarkii*, and have influenced their population genetic structure and diversity [9].

### Presumable Initial Entry Points

It has been reported that red swamp crayfish were introduced to Nanjing, China from Japan in 1929 [9], [22]. Our data showed that *P. clarkii* populations collected around Nanjing have a closer relationship with the Japanese one, and that Nanjing was probably the initial entry point of introduction of *P. clarkii*. First, the data on haplotype frequency showed that Hap_C1 and Hap_C2 were mainly distributed among *P. clarkii* populations collected in China and Japan, but Hap_C3, Hap_C4, Hap_C5 and Hap_C6 were only detected in *P. clarkii* population in the USA (Figure S1A, S2A). Similarly, Hap_S1 and Hap_S2 were mainly distributed among *P. clarkii* populations collected in China and Japan while the haplotype 3 (Hap_S3) was only detected in the *P. clarkii* population in the USA (Figure S1B, S2B). Second, microsatellite data showed that the population from Baguazhou township, Nanjing, had the highest genetic diversity among the populations analyzed in China (Table S1). Third, the phylogenetic tree showed that populations in NanJing (BGt and XG), or around Nanjing (CJr, NT and NB,), were clustered first with the foreign populations ((Figure 1, Figure S3). Fourth, the Bayesian assignment also indicated that most individuals from populations in Nanjing or around Nanjing (XG, BGt, NB and CJr) and foreign populations (Sa and Lo) were assigned to the same genetic cluster (Figure 2). Therefore, the results of this study further demonstrated that *P. clarkii* were introduced from Japan, Nanjing was probably the intial place of introduction of *P. clarkii*, and suburbs of Nanjing (such as Xiaguan District and Baguazhou township) were the presumable initial entry points. And then, they started dispersing in the vicinity of Jiangsu province and along the Changjiang river basin. Nevertheless, of course more genetic data from source area need to be collected for investigating the initial entry point for *P. clarkii* invasion.

### Single or Multiple Introductions?

It is believed that *P. clarkii* may have been introduced only once from Japan to Nanjing in 1929 [9], [22]. However, our genetic analyses supported that *P. clarkii* was likely introduced from multiple events. So, we speculated that unobserved multiple introduction events or cryptic invasions might exist.

The high diversity of invasive species population could be caused by multiple introductions from one or more places [23], [24], or a single introduction of a large number of individuals from different populations [24], [25]. However, the genetic diversity of invasive species populations was lower than that of the origin population after experiencing bottleneck and founder effect if the invaders were introduced only once from origin place [26], [27]. If the invasion species were derived from multiple introductions, their population genetic diversity would not necessarily be lower than that of the origin place. This is because multiple introductions could help the invasive population to maintain enough variations, even new genotypes occurring which are owed to inbreeding among populations derived from reproductive isolation in the origin place [28], [29]. For example, the genetic diversity and heritable phenotypic variation of the exotic grass, *Phalaris arundinacea*, in North America were higher than those in the place of origin in Europe [30]. In this study, the *P. clarkii* populations in China generally showed high genetic diversity. However, it was found that even if Baguazhou township, Nanjing, showed the highest allelic and gene diversity among the populations investigated in China by analysis of microsatellites, mitochondrial COI and 16S rRNA sequences. Thus, some unobserved multiple introduction events and cryptic invasions could not be excluded, although some documents reported that *P. clarkii* may have been introduced only once from Japan. As the Bayesian assignment showed, surprisingly, that populations NB, XG, BGt and CJr, and two more populations from Japan and the USA, were clustered together with extremely high membership coefficients. However, the other populations from China were grouped into another six genetic clusters. If Nanjing was the only initial entry point and only one introduction event occurred in the 1920s, all or almost all genetic variation should be detected in the area surrounding Nanjing. Here, there might be two explanations for the results of assignment: (i) multiple introductions which might generate divergent invasive populations [19], and/or other unintentional introductions in China might exist; (ii) this species might have experienced rapid evolutionary and/or genetic changes [20] associated with novel environments and/or aquaculture breeding.

### High Genetic Diversity and the Implications for Invasion Success

Some exotic invasions succeed despite founder effects, and consequent low genetic diversity was often due to the invasive populations experiencing bottlenecks and genetic drift [31]. However, the *P. clarkii* populations in China had very high diversity as shown in the present study. Other studies on *P. clarkii* in China have also showed similar results [5], [10]. Moreover, similar cases of high diversity and structure of introduced populations in Europe have been documented [23].

Generally, the idea that increased genetic diversity contributes to invasion success presupposes that evolution enhances invasions, and bottlenecks during invasion limit the adaptive evolution of fitness-related traits [32]. Evolutionary biologists postulated that genetic variation and evolution might play an important role in the success of invading species [33]. However, the high genetic diversity of invasive species may be caused by hybridization and variation after successfully invading a new environment [30]. Some studies have shown that putatively adaptive traits have evolved in introduced populations, and sometimes quite rapidly [34], [35]. A hypothesis of increased genetic diversity with some successful invaders putatively retaining adaptive variation is not necessarily inconsistent with the presence of genetic bottlenecks; this is because founding events were not only to be expected to eliminate all variation, but also because many fitness-related traits do not lose variation as quickly as do individual loci [32], [36]. In this study, AMOVA showed that a lot of genetic variation occurred among and within the *P. clarkii* populations (Table 1).

Multiple introductions or donors can rescue invaders from losses in diversity [32]. Intra-specific admixture from multiple native source regions could help introduced populations overcome founder effects and generate novel genetic substrates for selection in introduced ranges [37], [38]. In some cases, admixture from multiple sources has been shown to lead to significant variation in morphology and life-history traits between introduced populations, with potentially important implications for invasion success [38], [39]. Some novel differentiation could arise even in the case where it seems highly unlikely that it favored the genetic diversity of the species which has a potential global distribution [1].

In conclusion, the *P. clarkii* founder population in China might have been derived from Japan. *P. clarkii* populations in China have relatively high genetic diversity. Numerous factors likely facilitated invasion success of *P. clarkii*, such as high genetic diversity, adaptive variation, aquaculture activity and some ecological factors, and the absence of predators in China.

## Materials and Methods

### Sampling, DNA Isolation and Microsatellite Genotyping

We focused on the alleged initial places of introduction, and areas of major cultivation and major river basins where *P. clarkii* were distributed in China. A total of 35 sites were selected from China and one site each from the USA and Japan (Figure 1, Table S1). The sites in China are located in an open, abandoned field and no specific permit is required for the described field studies. The sites from Japan and the USA were kindly provided by Dr. Jian Gao from the Kagoshima University and Dr. Dan Wang from the Ohio State University South Centers.

Muscle cuts of 48 individuals were sampled at each site and were stored in 95% ethanol at −20°C for DNA extraction. Genomic DNA was isolated using the ammonium acetate method [40], and was stored at −20°C for subsequent experiments. Each sample was genotyped using methods as described by Li *et al.* [40] at 12 microsatellite loci: PcLG-03, PcLG-04, PcLG-07, PcLG-09, PcLG-10, PcLG-13, PcLG-15, PcLG-17, PcLG-29, PcLG-32 and PcLG-48 [41] and PcL24 [3].

### Mitochondrial COI and 16S rRNA Amplification

Six to ten DNA samples were randomly selected from each sampling site to amplify the partial mitochondrial COI and 16S rRNA fragments (Table S1). The partial COI gene fragments were amplified using primers LCO 1490 and HCO 2198 [42] under thermocycling conditions: 94°C for 5 min followed by 35 cycles of 94°C for 30 s, 50°C for 45 s and 72°C for 1 min with a final extension at 72°C for 10 min. The 16S rRNA fragments were amplified with the primers 1471 and 1472 [43], and the thermocycling conditions comprised 35 cycles of 30 s at 94°C, 1 min at 48°C and 1 min at 72°C. PCR products were directly sent to Invitrogen Biotech (Shanghai, China) Co., Ltd or Sangon Biotech (Shanghai, China) Co., Ltd. for sequencing.

### Data Analysis

Based on microsatellite data, the number of alleles (*Na*), number of effective alleles (*Ne*), the observed heterozygosity (*H_O_*) and the expected heterozygosity (*He*) were calculated using the program POPGENE version 1.31 [44]. Deviations from Hardy-Weinberg Equilibrium (HWE) using Markov chain reaction were performed by Genepop 4.0.10 [45], [46]. To analyze clusters of 37 *P. clakii* populations, the Neighbour-Joining and UPGMA dendrograms for populations based on the Nei’s genetic distance matrix were constructed using the software MEGA 5.05 [47].

Structure version 2.3.1 [15], [48] was used to assign individuals to populations by a Bayesian approach using microsatellite genotype data, with the power to acknowledge that a sample site does not necessarily represent a true genetic population. Structure 2.3.1 was run using the correlated allele frequencies model with a burn-in period of 100,000 MCMC (Markov Chain Monte Carlo) steps followed by 1,000,000 iterations, with eight independent runs conducted to assess the consistency of the results across runs. All iterations were run with the admixture model, using a Bayesian approach with prior distributions of model parameters. Bayesian methods assume that observations are randomly drawn from each cluster and that all potential source populations are predefined [49]. We used an *ad hoc* method for estimating the number of genetic clusters [16], [17]. ARLEQUIN v3.5.1.2 [50] was applied for analysis of molecular variance (AMOVA). Ratios of the variance components could be then used to define population structure.

Correlation between genetic distance and geographic distance was assessed using IBDWS version 3.21 [51]. Significance of the analysis was examined using Mantel tests as implanted in software IBDWS.

Moreover, based on mitochondrial COI and 16S rRNA sequences, the variation sites, parsimony informative sites, number of haplotype, and nucleotide diversity were determined by using the soft DnaSP 5.10 [52]. The neutral test (Fu’*Fs* and Li’s tests) was also considered by DnaSP 5.10. Analysis of molecular variance (AMOVA) was conducted in ARLEQUIN v3.5.1.2 to calculate the variance components and significance levels of variation within a population and among populations. Relationships among mtDNA COI and 16S rRNA haplotypes were examined respectively using a statistical parsimony haplotype network generated at the 95% connection limit with TCS version 1.21 [53].

## Supporting Information

Figure S1 **One sequence of haplotypes and distribution of all haplotypes obtained from mtDNA COI and 16S rRNA sequences.**
(PDF)

Figure S2 **Statistical parsimony networks of mtDNA COI (A) and 16S rRNA (B) sequences for** ***P. clarkii*** **samples.**
(PDF)

Figure S3 **UPGMA dendrogram of 37** ***P. clarkii*** **populations based on Nei’s (1972) genetic distance (** ***D*** **).**
(PDF)

Table S1 **List of the populations of** ***P. clarkii*** **studied indicating the location, their country of origin, geographical position of sampling sites, genetic diversity at 12 microsatellite loci, and haplotype diversity at mtDNA COI and 16S rRNA sequences.**
(PDF)
