# Supplementary material for: Population Genetic Structure and Post-Establishment Dispersal Patterns of the Red Swamp Crayfish Procambarus Clarkii in China
Source: PLoS One. 2012 Jul 10;7(7):e40652. doi: 10.1371/journal.pone.0040652 (PMC3393698; doi:10.1371/journal.pone.0040652)
Supplement: Figure S1 — One sequence of haplotypes and distribution of all haplotypes obtained from mtDNA COI and 16S rRNA sequences. (PDF) [file pone.0040652.s001.pdf]

**A**

Hap_C1 (637bp) :

| | | |
|---|---|---|
| 1 | GGTATAGTAGGAACTTCATTAAGAATGATTATTCGGGTGGAGTTAGGTCAACCAGGAAGATTAATTGGGGATGATCAGATCTATAATGTGGTAGTTACAGCTCATGCTTT | 110 |
| 111 | TGTAATAATTTTTTTTATAGTAATACCTATTATAATTGGTGGGTTTGGAAATTGATTAATTCCTTTAATATTAGGTGCTCCAGATATGGCTTTTCCTCGAATAAATAATA | 220 |
| 221 | TAAGGTTTTGATTACTTCCTTTTTCTTTGACTTTATTATTAACTAGGGGTATAGTTGAGAGAGGAGTTGGAACAGGGTGGACTGTTTATCCTCCTTTAGCTTCTGCTATT | 330 |
| 331 | GCTCATGCGGGAGCATCTGTAGATTTAGGTATTTTTTCTCTACATTTAGCAGGTGTATCTTCTATTTTAGGTTCAGTAAATTTTATAACAACTGCTATTAATATACGAAC | 440 |
| 441 | AGTAGGGATAACCATGGATCGAATGCCGTTATTTGTTTGATCAGTGTTTATTACTACTGTTTTATTATTATTATCTTTACCTGTGTTGGCAGGAGCTATTACTATATTAT | 550 |
| 551 | TAACAGATCGTAATCTAAATACTTCTTTTTTTGATCCAGCAGGGGGAGGGGATCCTATTTTATATCAGCATTTATTTTGATTTTTTG | 637 |

| | | | | | | | | | | | | | | | | | | | | | | | | | | | | | | | | |
|---|---|---|---|---|---|---|---|---|---|---|---|---|---|---|---|---|---|---|---|---|---|---|---|---|---|---|---|---|---|---|---|---|
| | | | 2 | 2 | 2 | 3 | 4 | 4 | 4 | 4 | 5 | 5 | 5 | 5 | 6 | | | | | | | | | | | | | | | | | |
| | 4 | 4 | 3 | 8 | 9 | 4 | 0 | 4 | 6 | 8 | 2 | 3 | 6 | 9 | 0 | SH | JX | WXb | NT | XB | WX | WJ | MAS | CH | HF | DY | NBp | ZX | JY | CQs | Sa | Lo |
| | 2 | 5 | 4 | 8 | 7 | 2 | 8 | 4 | 5 | 6 | 8 | 4 | 5 | 4 | 3 | | | | | | | | | | | | | | | | | |
| Hap_C1 | G | A | A | T | G | A | A | A | G | G | G | A | C | G | T | 4 | 2 | 3 | 1 | 4 | 3 | 8 | 8 | 6 | 6 | 5 | 1 | 6 | 3 | 6 | 6 | |
| Hap_C2 | . | . | . | . | A | . | . | . | A | . | . | . | . | A | . | 4 | 8 | 4 | 6 | 4 | 5 | | 2 | 2 | 1 | 5 | 7 | 4 | 7 | | 4 | |
| Hap_C3 | . | . | . | C | A | . | G | . | A | . | A | . | . | A | . | | | | | | | | | | | | | | | | | 7 |
| Hap_C4 | A | . | G | A | A | G | . | G | A | A | . | G | . | A | C | | | | | | | | | | | | | | | | | 1 |
| Hap_C5 | A | G | G | A | A | G | . | G | A | . | . | G | . | A | C | | | | | | | | | | | | | | | | | 1 |
| Hap_C6 | . | . | . | C | A | . | . | . | A | . | A | . | T | A | . | | | | | | | | | | | | | | | | | 1 |

Note: The haplotype of the population NB, XYc, XYw, XG, BGt, CJr, SLt, PYL, NCyl, NHL, YNL, XT, QJ, LZL, HHL, CHL, YJ, NX, DTL and DTLs was Hap_C2.

**B**

Hap_S1 (509bp) :

| | | |
|---|---|---|
| 1 | ACCTGCCCATTGGGAACTAAAAGGCCGCGGTATTATGACCGTGCAAAGGTAGCATAATCATTAGTTTTTTAATTGAAGGCTAGAATGAATGGTTGGACAAGAAATAATCT | 110 |
| 111 | GTCTTAAATTAATATATTGAATTTAACTTTTAAGTGAAAAGGCTTAAATAATCTGGAGGGACGATAAGACCCTATAAAACTTTATATTTATAATATAGTAGTTAGTTTTA | 220 |
| 221 | TTTAAGGGTATTATTTTAGAGTATTTGGTTGGGGTGACAAGGATAAAATATTAAATAACTGTCTTTTTTTTTT-ACAGTGATGTTTGGTTTAATGATCCTAAAAGGGATT | 330 |
| 331 | AAAGATTAAGTTACTTTAGGGATAACAGCGTAATTTTCTTTAAGAGTTCTTATCGACAAGAAAGTTTGCGACCTCGATGTTGAATTAAAAGTTCTTTATAGAGTAGAGAC | 440 |
| 441 | TATAATAGAAGGTCTGTTCGACCTTTAAAATTTTACATGATTTGAGTTCAGACCGGTGTAAGCCAGGTT | 509 |

| | | | | | | | | | | | | | | | | | | | | |
|---|---|---|---|---|---|---|---|---|---|---|---|---|---|---|---|---|---|---|---|---|
| | | 2 | 2 | | | | | | | | | | | | | | | | | |
| | 9 | 2 | 9 | SH | JX | WXb | NT | XB | WX | WJ | MAS | CH | HF | DY | NBp | CQs | ZX | JY | Sa | Lo |
| | 6 | 5 | 4 | | | | | | | | | | | | | | | | | |
| Hap_S1 | A | A | - | 3 | 2 | 2 | 1 | 3 | 3 | 7 | 6 | 6 | 7 | 3 | 1 | 8 | 5 | 2 | 5 | 2 |
| Hap_S2 | G | . | T | 4 | 6 | 5 | 5 | 4 | 5 | | 2 | 2 | 1 | 5 | 7 | | 2 | 6 | 3 | |
| Hap_S3 | G | G | - | | | | | | | | | | | | | | | | | 6 |

Note：The haplotype of the population NB, XYc, XYw, XG, BGt, CJr, SLt, PYL, NCyl, NHL, YNL, XT, QJ, LZL, HHL, CHL, YJ, NX, DTL and DTLs was Hap_S2.

**Figure S1. One sequence of haplotypes and distribution of all haplotypes obtained.** A, Haplotype C1 (Hap_C) sequence and distribution of haplotypes in COI sequences of *P. clarkii*; B, Haplotype S1 (Hap_S) sequence and distribution of haplotypes in 16S rDNA sequences of *P. clarkii*.
