# Supplementary material for: Population Genetic Structure and Post-Establishment Dispersal Patterns of the Red Swamp Crayfish Procambarus Clarkii in China
Source: PLoS One. 2012 Jul 10;7(7):e40652. doi: 10.1371/journal.pone.0040652 (PMC3393698; doi:10.1371/journal.pone.0040652)
Supplement: Figure S2 — Statistical parsimony networks of mtDNA COI (A) and 16S rRNA (B) sequences for P. clarkii samples. (PDF) [file pone.0040652.s002.pdf]

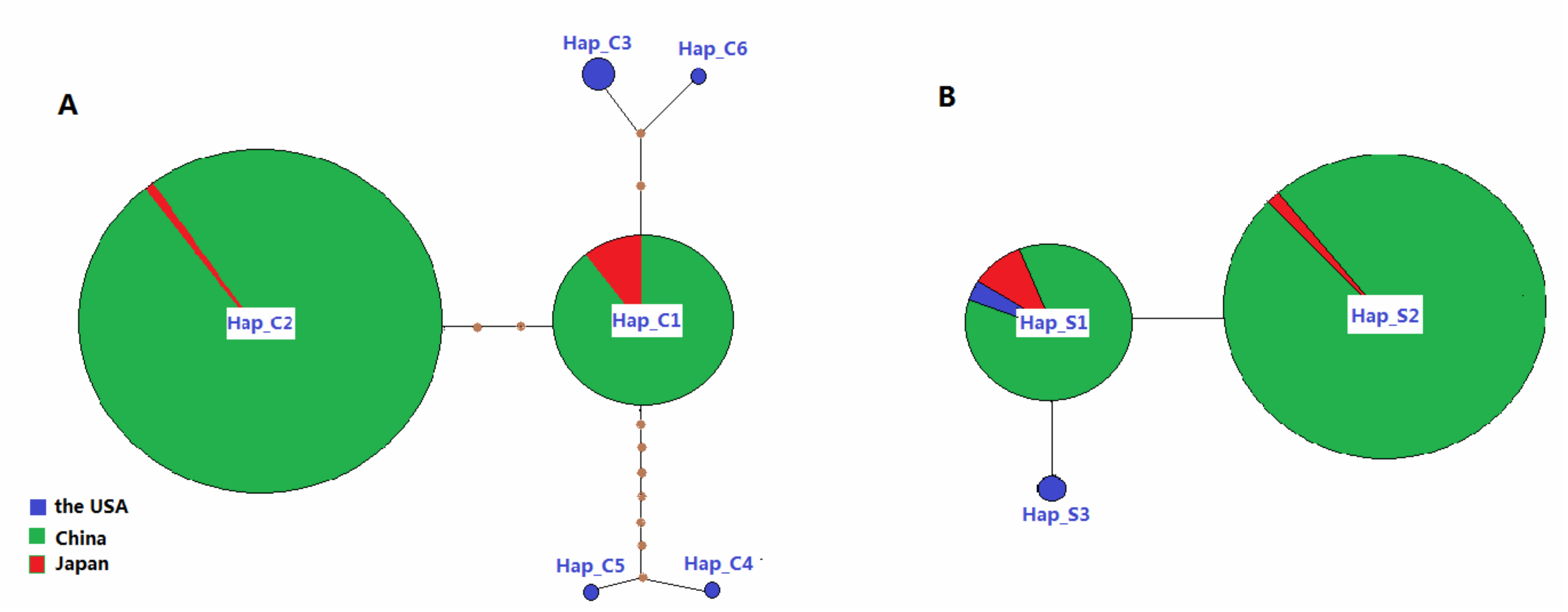

Figure S2. Statistical parsimony networks of mtDNA COI (A) and 16S rDNA (B) sequences for *P. clarkii* samples. Small brown dots represented missing haplotypes. Haplotypes were painted according to different geographical regions from which the sample was collected. Circle size was proportional to observed haplotype frequency.
