# Supplementary figures and images for: Population Genetic Structure and Post-Establishment Dispersal Patterns of the Red Swamp Crayfish Procambarus Clarkii in China
Source: PLoS One. 2012 Jul 10;7(7):e40652. doi: 10.1371/journal.pone.0040652 (PMC3393698; doi:10.1371/journal.pone.0040652)

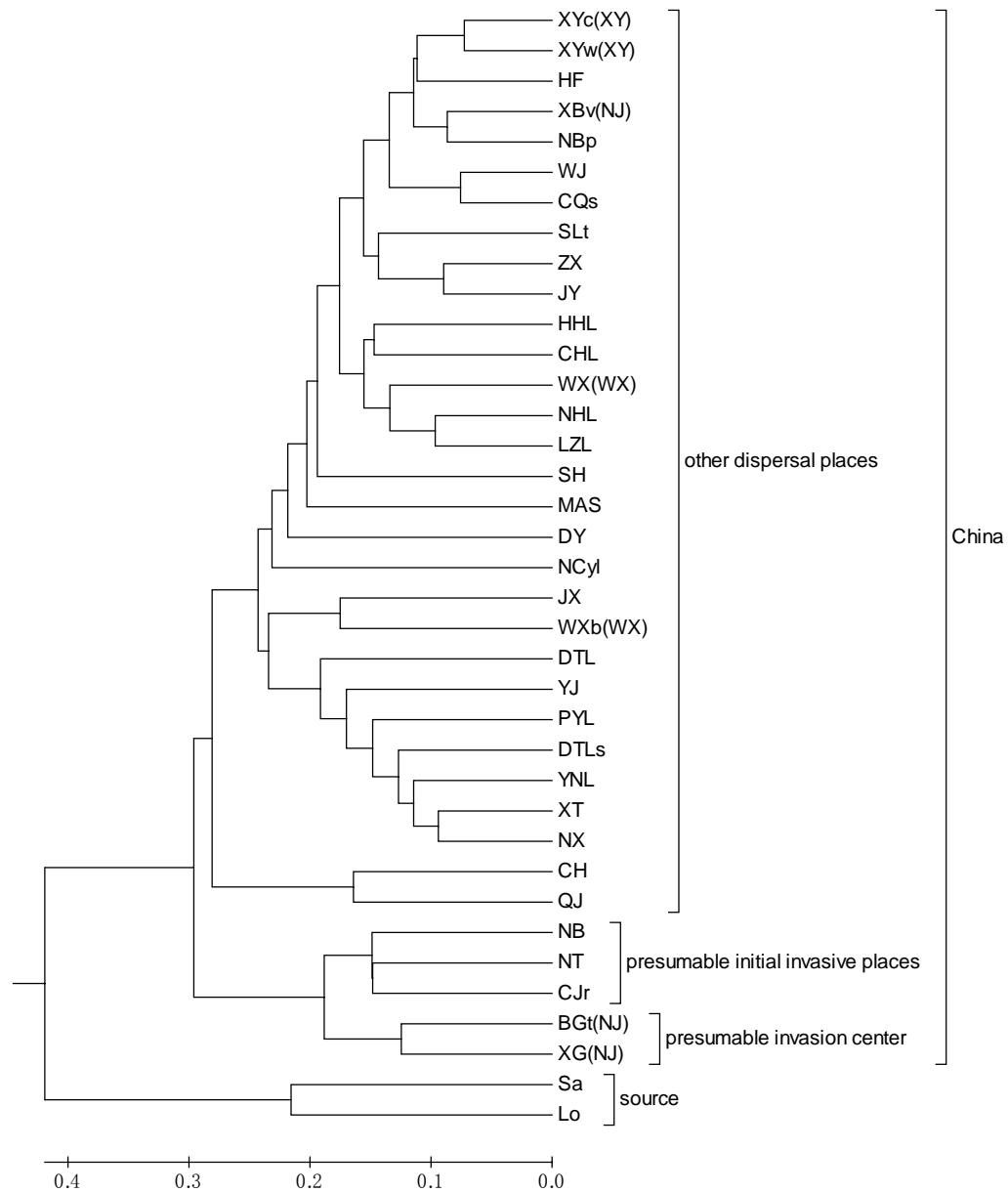

**Figure S3. UPGMA dendrogram of 37 *P. clarkii* populations based on Nei's (1972) genetic distance ( $D$ ).**

Supplement: Figure S3 — UPGMA dendrogram of 37 P. clarkii populations based on Nei’s (1972) genetic distance ( D ). (PDF) [file pone.0040652.s003.pdf]
