# Supplementary material for: Population Genetic Structure and Post-Establishment Dispersal Patterns of the Red Swamp Crayfish Procambarus Clarkii in China
Source: PLoS One. 2012 Jul 10;7(7):e40652. doi: 10.1371/journal.pone.0040652 (PMC3393698; doi:10.1371/journal.pone.0040652)
Supplement: Table S1 — List of the populations of P. clarkii studied indicating the location, their country of origin, geographical position of sampling sites, genetic diversity at 12 microsatellite loci, and haplotype diversity at mtDNA COI and 16S rRNA sequences. (PDF) [file pone.0040652.s004.pdf]

**Table S1.** List of the populations of *P. clarkii* studied indicating the location, their country of origin, geographical position of sampling sites, genetic diversity at 12 microsatellite loci, and haplotype diversity at mtDNA COI and 16S rDNA sequences.

| Code | Location (Country) | Longitude | Latitude | Microsatellite genotyping | | | | | | COI sequencing | | | 16S rDNA sequencing | | |
|---|---|---|---|---|---|---|---|---|---|---|---|---|---|---|---|
| | | | | Microsat (N) | *Na* | *Ne* | *Ho* | *He* | *PIC* | COI (N/H) | *Hd* | *Pi* | 16S rDNA (N/H) | *Hd* | *Pi* |
| SH | Shanghai (China) | 121.23°E | 31.03°N | (48) | 11.75 | 6.48 | 0.7887 | 0.8214 | 0.7903 | (8/2) | 0.571 | 0.0027 | (7/2) | 0.571 | 0.0011 |
| NB | Ningbo (China) | 121.55°E | 29.88°N | (48) | 10.42 | 5.36 | 0.5880 | 0.8068 | 0.7738 | (7/1) | 0.000 | 0.0000 | (8/1) | 0.000 | 0.0000 |
| JX | Jiaxing (China) | 120.77°E | 30.75°N | (48) | 8.08 | 5.41 | 0.6632 | 0.7860 | 0.7441 | (10/2) | 0.356 | 0.0017 | (8/2) | 0.429 | 0.0008 |
| XYc | Xuyi-culture (China) | 118.50°E | 33.00°N | (48) | 9.50 | 5.37 | 0.7425 | 0.7998 | 0.7623 | (7/1) | 0.000 | 0.0000 | (7/1) | 0.000 | 0.0000 |
| XYw | Xuyi-wild (China) | 118.42°E | 33.03°N | (48) | 9.67 | 5.45 | 0.7342 | 0.7933 | 0.7542 | (10/1) | 0.000 | 0.0000 | (8/1) | 0.000 | 0.0000 |
| WXb | Binhu, Wuxi (China) | 120.28°E | 31.52°N | (48) | 9.25 | 5.78 | 0.6416 | 0.7978 | 0.7578 | (7/2) | 0.571 | 0.0027 | (7/2) | 0.476 | 0.0009 |
| NT | Nantong (China) | 120.87°E | 32.02°N | (48) | 10.08 | 5.58 | 0.6181 | 0.8211 | 0.7931 | (7/2) | 0.286 | 0.0014 | (6/2) | 0.333 | 0.0007 |
| XG | Xiaguan district (Nanjing,China) | 118.75°E | 32.08°N | (48) | 10.08 | 6.23 | 0.5602 | 0.8151 | 0.7792 | (10/1) | 0.000 | 0.0000 | (8/1) | 0.000 | 0.0000 |
| XBv | Xiaba village (Nanjing,China) | 118.87°E | 32.20°N | (48) | 10.17 | 5.80 | 0.7836 | 0.7877 | 0.7557 | (8/2) | 0.571 | 0.0027 | (7/2) | 0.571 | 0.0011 |
| BGt | Baguazhou | 118.82°E | 32.17°N | (48) | 11.08 | 6.73 | 0.6285 | 0.8413 | 0.8124 | (8/1) | 0.000 | 0.0000 | (8/1) | 0.000 | 0.0000 |

| | | | | | | | | | | | | | | | | |
|---|---|---|---|---|---|---|---|---|---|---|---|---|---|---|---|---|
| | township (Nanjing,China) | | | | | | | | | | | | | | | |
| WX | Wuxi (China) | 120.30°E | 31.57°N | (48) | 10.00 | 5.59 | 0.7786 | 0.7889 | 0.7536 | (8/2) | 0.536 | 0.0025 | (8/2) | 0.536 | 0.0011 |
| WJ | Wangjiang (China) | 116.70°E | 30.12°N | (48) | 10.42 | 6.18 | 0.8129 | 0.8025 | 0.7672 | (8/1) | 0.000 | 0.0000 | (7/1) | 0.000 | 0.0000 |
| MAS | Maanshan (China) | 118.50°E | 31.55°N | (48) | 9.50 | 5.60 | 0.6413 | 0.7771 | 0.7359 | (10/2) | 0.356 | 0.0017 | (8/2) | 0.429 | 0.0008 |
| CJr | Guangfengwei section of Changjiang river (China) | 116.87°E | 30.12°N | (48) | 10.00 | 5.65 | 0.5777 | 0.7984 | 0.7621 | (8/1) | 0.000 | 0.0000 | (8/1) | 0.000 | 0.0000 |
| CH | Chaohu (China) | 117.87°E | 31.62°N | (48) | 10.42 | 5.74 | 0.7413 | 0.7786 | 0.7439 | (8/2) | 0.429 | 0.0020 | (8/2) | 0.429 | 0.0008 |
| HF | Hefei (China) | 117.23°E | 31.82°N | (48) | 8.75 | 4.88 | 0.6656 | 0.7623 | 0.7222 | (7/2) | 0.286 | 0.0014 | (8/2) | 0.250 | 0.0005 |
| DY | Dingyuan (China) | 117.83°E | 32.28°N | (48) | 8.75 | 4.84 | 0.6285 | 0.7428 | 0.7018 | (10/2) | 0.556 | 0.0026 | (8/2) | 0.536 | 0.0011 |
| SLt | Sanli township (China) | 116.22°E | 29.75°N | (48) | 10.33 | 5.77 | 0.7007 | 0.7847 | 0.7497 | (8/1) | 0.000 | 0.0000 | (8/1) | 0.000 | 0.0000 |
| NBp | Nanbei Port (China) | 116.17°E | 29.72°N | (48) | 10.67 | 5.47 | 0.6991 | 0.7969 | 0.7628 | (8/2) | 0.250 | 0.0012 | (8/2) | 0.250 | 0.0005 |
| PYL | Poyang lake (China) | 116.43°E | 28.87°N | (48) | 8.9167 | 4.89 | 0.5890 | 0.7448 | 0.7086 | (10/1) | 0.000 | 0.0000 | (7/1) | 0.000 | 0.0000 |
| NCyl | Youlan, Nanchang (China) | 116.12°E | 28.52°N | (48) | 9.75 | 5.80 | 0.6742 | 0.7845 | 0.7433 | (6/1) | 0.000 | 0.0000 | (7/1) | 0.000 | 0.0000 |

| | | | | | | | | | | | | | | | | |
|---|---|---|---|---|---|---|---|---|---|---|---|---|---|---|---|---|
| NHL | Nanhu lake (China) | 114.03°E | 30.02°N | (48) | 10.75 | 5.96 | 0.7774 | 0.8194 | 0.7883 | (8/1) | 0.000 | 0.0000 | (8/1) | 0.000 | 0.0000 |
| YNL | Yuni lake (China) | 112.20°E | 30.00°N | (48) | 10.58 | 5.84 | 0.7361 | 0.8224 | 0.7905 | (7/1) | 0.000 | 0.0000 | (8/1) | 0.000 | 0.0000 |
| XT | Xiantao (China) | 113.40°E | 30.30°N | (48) | 9.17 | 5.28 | 0.7096 | 0.7940 | 0.7559 | (8/1) | 0.000 | 0.0000 | (7/1) | 0.000 | 0.0000 |
| QJ | Qianjiang (China) | 112.60°E | 30.40°N | (48) | 9.92 | 5.50 | 0.6764 | 0.7892 | 0.7535 | (10/1) | 0.000 | 0.0000 | (8/1) | 0.000 | 0.0000 |
| LZL | Liangzi lake (China) | 114.00°E | 30.00°N | (48) | 9.67 | 5.64 | 0.6715 | 0.8031 | 0.7658 | (10/1) | 0.000 | 0.0000 | (8/1) | 0.000 | 0.0000 |
| HHL | Honghu lake (China) | 113.40°E | 29.70°N | (48) | 10.67 | 5.85 | 0.7466 | 0.7924 | 0.7559 | (8/1) | 0.000 | 0.0000 | (8/1) | 0.000 | 0.0000 |
| CHL | Changhu lake (China) | 112.10°E | 30.30°N | (48) | 9.58 | 4.81 | 0.5875 | 0.7482 | 0.7089 | (6/1) | 0.000 | 0.0000 | (7/1) | 0.000 | 0.0000 |
| YJ | Yuanjiang (China) | 112.37°E | 28.85°N | (48) | 7.92 | 4.53 | 0.5942 | 0.7584 | 0.7162 | (10/1) | 0.000 | 0.0000 | (6/1) | 0.000 | 0.0000 |
| NX | Ningxiang (China) | 112.55°E | 28.28°N | (48) | 8.00 | 4.41 | 0.5569 | 0.7400 | 0.6944 | (8/1) | 0.000 | 0.0000 | (8/1) | 0.000 | 0.0000 |
| DTL | Dongting lake (China) | 113.02°E | 29.30°N | (48) | 8.67 | 5.0804 | 0.6782 | 0.7860 | 0.7459 | (10/1) | 0.000 | 0.0000 | (10/1) | 0.000 | 0.0000 |
| DTLs | Dongting lakeside (China) | 113.13°E | 29.35°N | (48) | 10.25 | 5.87 | 0.7038 | 0.8170 | 0.7850 | (9/1) | 0.000 | 0.0000 | (8/1) | 0.000 | 0.0000 |
| CQs | Chongqing suburb (China) | 106.53°E | 29.55°N | (48) | 9.00 | 4.88 | 0.7182 | 0.7593 | 0.7123 | (6/1) | 0.000 | 0.0000 | (8/1) | 0.000 | 0.0000 |
| ZX | Zhongxian (China) | 108.03°E | 30.28°N | (48) | 6.42 | 3.68 | 0.5748 | 0.7002 | 0.6553 | (10/2) | 0.533 | 0.0025 | (7/2) | 0.476 | 0.0009 |

| | | | | | | | | | | | | | | | |
|---|---|---|---|---|---|---|---|---|---|---|---|---|---|---|---|
| JY | Jianyang (China) | 104.55°E | 30.38°N | (48) | 7.83 | 4.34 | 0.6109 | 0.7512 | 0.7113 | (10/2) | 0.467 | 0.0022 | (8/2) | 0.429 | 0.0008 |
| Sa | Saitama (Japan) | 139.65°E | 35.85°N | (48) | 14.33 | 9.37 | 0.6283 | 0.8873 | 0.8667 | (10/2) | 0.533 | 0.0025 | (8/2) | 0.536 | 0.0011 |
| Lo | Louisiana (the USA) | 93.26°W | 29.87°N | (48) | 14.58 | 8.81 | 0.6465 | 0.8799 | 0.8579 | (10/4) | 0.533 | 0.0062 | (8/2) | 0.429 | 0.0008 |

Microsat = microsatellite, N = sample size, *H* = number of haplotypes, *Na* = mean number of alleles, *Ne* = mean number of effective alleles, *PIC* = mean polymorphism information content, *H*o = mean observed heterozygosity, *He* = mean expected heterozygosity, *Hd* = haplotype diversity, and *Pi* = nucleotide diversity.
